# Integration of a *FT* expression cassette into CRISPR/Cas9 construct enables fast generation and easy identification of transgene-free mutants in Arabidopsis

**DOI:** 10.1371/journal.pone.0218583

**Published:** 2019-09-23

**Authors:** Yuxin Cheng, Na Zhang, Saddam Hussain, Sajjad Ahmed, Wenting Yang, Shucai Wang

**Affiliations:** 1 Key Laboratory of Molecular Epigenetics of MOE, Northeast Normal University, Changchun, Jilin, China; 2 College of Life Science, Linyi University, Linyi, Shandong, China; Instituto de Biologia Molecular y Celular de Plantas, SPAIN

## Abstract

The CRISPR/Cas9 genome editing technique has been widely used to generate transgene-free mutants in different plant species. Several different methods including fluorescence marker-assisted visual screen of transgene-free mutants and programmed self-elimination of CRISPR/Cas9 construct have been used to increase the efficiency of genome edited transgene-free mutant isolation, but the overall time length required to obtain transgene-free mutants has remained unchanged in these methods. We report here a method for fast generation and easy identification of transgene-free mutants in Arabidopsis. By generating and using a single *FT* expression cassette-containing CRISPR/Cas9 construct, we targeted two sites of the *AITR1* gene. We obtained many early bolting plants in T1 generation, and found that about two thirds of these plants have detectable mutations. We then analyzed T2 generations of two representative lines of genome edited early bolting T1 plants, and identified plants without early bolting phenotype, i.e., transgene-free plants, for both lines. Further more, *aitr1* homozygous mutants were successful obtained for both lines from these transgene-free plants. Taken together, these results suggest that the method described here enables fast generation, and at the mean time, easy identification of transgene-free mutants in plants.

## Introduction

Shortly after the reports that CRISPR (clustered regularly interspaced short palindromic repeats) RNA-guided Cas9 (CRISPR-associated protein 9) endonuclease is able to cleave double-stranded DNA, and can be used to generate mutations in eukaryotic cells [1,2], CRISPR/Cas9 mediated genome-editing has been successful used for gene editing to generate mutations in several different plants including the model plant Arabidopsis and crops such as rice, tobacco and wheat [3–5]. Since then, the CRISPR/Cas9 genome-editing techniques including the double-stranded DNA cleaving based editing and the nucleotide substitution based editing have been widely used to generate mutations in different plant species, in some cases, to improve agronomic traits such as yield, quality and biotic and abiotic stress tolerances [6–12]. Thanks to their high efficiency in genome editing and the use of engineered Cas9 variants with expanded target space, CRISPR/Cas9 genome-editing systems have brought a bright future for plant breeding [6,12–14].

The presence of Cas9 T-DNA in CRISPR/Cas9 genome-edited mutants may affect the phenotypic stability and heritability of the mutation, and transgene-free is likely required for commercial application of CRISPR/Cas9 genome-edited crops [7, 10]. Therefore, isolation of transgene-free mutants is one of the most important steps for generation mutants by using CRISPR/Cas9 genome-editing. However, isolation of transgene-free mutants by using the traditional genetic segregation and backcross based genotyping is time consuming and laborious [7,8,10]. To improve the efficiency in transgene-free mutant isolation from CRISPR/Cas9 genome edited plants, a few different methods have been established [6–8,10]. These methods include the fluorescence maker-assisted selection, which allows to isolate transgene-free mutants based on the observation of the absence of fluorescence in seeds produced by transgenic plants [7]; the active interference element mediated selection, which allows herbicide-dependent isolation of transgene-free plants [8]; and the programmed self-elimination system, which allows only transgene-free male gametophytes to produce seeds [13]. All these methods greatly reduced workload for transgene-free mutant isolation. However, the overall time length required for the whole process of mutant generation, from plant transformation, to mutant identification, and then transgene-free mutant isolation remained largely unchanged.

Appropriate flowering time is critical for successful sexual reproduction in flowering plants [15]. In order to achieve sexual reproduction successful, flowering plants need to sense and respond to environmental stimuli appropriately, and then integrate the environmental information with endogenous signals to make transit from vegetative growth to flowering [16–18]. Accumulated evidence suggest that flowering time in Arabidopsis is controlled by several different regulators, including CO (CONSTANS), SOC1 (SUPRESSOR OF CONSTANS OVEREXPRESSION1), FLM (FLOWERING LOCUS M), FLC, FLK (FLOWERING LOOCUS K HOMOLOGY (KH) DOMAIN), VRN2 (VERNALIZATION 2), MAF2 (MADS AFFECTING FLOWERING 2) and FT (FLOWERING LOCUS T) [15,17,19–24]. Among them, FLC and CO are major regulators involved in vernalization and photoperiod, the two most important environmental stimuli that control the switch from vegetative growth to flowering, respectively, and functioned immediately upstream of FT to regulate the switch [15–17].

FT is the key positive regulator of flowering in Arabidopsis, and at least some of the FT homologues in other plant species including medicago, rice, soybean and trees like poplar and pear also function as an activator of flowering [25–28]. Due to its important role in flowering promotion, FT has been successfully used to reducing juvenile phase of many plants, therefore accelerated the process for plant breeding [15,27,29].

Considering that early flowering phenotype caused by overexpression of *FT* in plants is easy to observe, and the resulted short life cycle will accelerate mutant generation, integration of a FT expression cassette into CRISPR/Cas9 may enable fast generation and easy identification of transgene-free mutants in plants. In this study, we introduced a *GmFT2a* expression cassette into the *pHEE* CRISPR/Cas9 vector, and inserted two *sgRNA* expression cassettes to target the *AITR1* (*ABA induced transcription repressor1*) gene, which encodes a novel ABA signaling and abiotic stress tolerance regulating transcription factor in Arabidopsis [30]. We successfully obtained detectable mutations in *AITR1* in early bolting T1 Arabidopsis transgenic plants, and obtained homozygous transgene-free *aitr1* mutants from T2 plants with normal bolting phenotypes.

## Materials and methods

### Plant materials and growth conditions

The Columbia ecotype ‘Col-0’ (Col) Arabidopsis (*Arabidopsis thaliana*) was used for plant transformation, and as controls for bolting time assays.

Seeds of Col wild type were germinated in soil pots, and grown in a growth room. T1 transgenic plants were selected by plating T1 seeds on antibiotic-containing 1/2 MS plates. Transgenic seedlings were transferred into soil pots, and grown in a growth room. As a control, seeds of Col wild type were germinated on 1/2MS plates, seedlings were transferred into soil pots and grown in a growth room. The growth conditions in the growth room have been described previously [31,32].

### Constructs

The *pHEE* CRISPR/Cas9 vector has been described previously [33]. To insert the FT expression cassette into *pHEE* to generate the *pHEE-FT* vector, the full-length open reading frame (ORF) sequence of *GmFT2a* was synthesized and cloned into *pUC19* under the control of the double *35S* promoter, and terminated by *nos* [34]. The sequence of the *35S*:*GmFT2a-nos* cassette was then amplified by PCR, and cloned into the *pHEE* vector at the *Pme*1 site by using Gibson assembly. The primers used to amplify *35S*:*GmFT2a-nos* from the *pUC19* construct were: 5’-CCTGTCAAACACTGATAGTTTGTCGACTCTAGAGGATCC-3’ and 5’-GTCGTTTCCCGCCTTCAGTTTACGACGGCCAGTGAATTC -3’.

To generate CRISPR/Cas9 construct for genome editing of *AITR1*, appropriate target sequences were first identified by scanning coding sequence of *AITR1* on CRISPRscan (http://www.crisprscan.org/?page=sequence), and then evaluated on Cas-OFFinder (http://www.rgenome.net/cas-offinder/). Two target sequences were selected and used for editing *of AITR1*, 5’-GATCTGACTTGTCTGATGAT(CGG)-3’, and 5’-GGTGGCGGAGGAGGCAACGG(CGG)-3’. The sgRNA expression cassettes targeting *AITR1* were cloned into the *pHEE-FT* vector to generate *pHEE-FT-AITR1* construct by following the procedures described by Wang et al [34]. The primers used to insert the target sequences into sgRNA expression cassettes were, *DT1-BsF*, 5’-ATATATGGTCTCGATTGATCTGACTTGTCTGATGATGTT-3’, *DTI-F0*, 5’-T GATCTGACTTGTCTGATGATGTTTTAGAGCTAGAAATAGC-3’, *DT2-R0*, 5-AACCCGTTGCCTCCTCCGCCACCAATCTCTTAGTCGACTCTAC-3’, and *DT2-BsR*, 5’-ATTATTGGTCTCGAAACCCGTTGCCTCCTCCGCCACC-3’. The primers used for colony PCR and to sequence the sgRNA expression cassettes in the generated *pHEE-FT-AITR1* construct were, *U6-26-IDF*, 5’-TGTCCCAGGATTAGAATGATTAGGC-3’ and *U6-26-IDR*, 5’-AGCCCTCTTCTTTCGATCCATCAAC-3’.

### Plant transformation and transgenic plant selection

Col wild type plants have several mature flowers on the main inflorescence (~5-week-old) were used for transformation. The plants were transformed via Agrobacterium *GV3101* mediated floral dipping [35]. T1 seeds collected were germinated on 1/2 MS plates containing 30 μg/ml hygromycin and 100 μg/ml carbenicillin to select transgenic plants.

Bolting time of the T1 plants was observed, and gene editing status was examined by amplifying the coding sequence of *AITR1* and sequencing the PCR products obtained.

### Isolation of transgene-free homozygous mutants

T2 seeds were collected from selected plants that bolted early and have the *AITR1* gene edited. The seeds were then sown directly into soil pots. T2 plants with normal bolting time were selected, the absence of *Cas9* T-DNA insertion was confirmed by PCR amplification of *Cas9* fragment, and gene editing status was further examined/confirmed amplifying the coding sequence of *AITR1* and sequencing the PCR products obtained.

### DNA isolation and PCR

To examine the gene editing status of *AITR1*, DNA was isolated from the leaves of transgenic plants, and the coding sequence of *AITR1* was amplified by PCR. PCR products were recovered from gel and sequenced. The sequencing results obtained were then examined and aligned with the coding sequence of *AITR1* obtained from Phytozome (http://phytozome.jgi.doe.gov/pz/portal.html). The primers used for PCR amplification of *ACT2* and *AITR1* coding sequence and have been described previously [30,36].

To confirm the transgene-free status of the mutants obtained, DNA was isolated from leaves of T2 transgenic plants that have a normal bolting time, and *Cas9* gene fragment was amplified by PCR. The primers used for PCR amplification of *Cas9* were, *Cas9-F*, 5’-GGACAACGAGGAGAATGAGG-3’, and *Cas9-R*, 5’-TGTCTCGACCAGCTGCCTCTT-3’

### Bolting time assays

For the T1 plants, the date of bolting was recorded, and days after the transgenic seedlings were transferred into soil pots were calculated and used as the bolting time. Transferred Col wild type plants were used as controls.

For T2 plants, the date of bolting was recorded, and days to bolting after seed germination were calculated. Col wild type plants germinated and grown in soil pots were used as controls.

## Results

### Generation of a *FT* expression cassette-containing CRISPR/Cas9 construct

Overexpression of *FT* in plants is able to promote flowering, therefore shorten the juvenile phase of the transgenic plants even in trees [27]. If used in CRISPR/Cas9 mediated gene editing, the easily visible early bolting phenotype caused by overexpression of *FT* may serve as an assistant selection marker for easy identification of transgene-free mutants, whereas early flowering due to shortening of the juvenile phase may reduce the length of the overall time required for generating genome edited mutants, thereby providing a method for fast generation and easy identification of genome edited transgene-free mutants.

FT promotes flowering in many plant species including the model plants Arabidopsis, crops such as rice and soybean, and fruit trees such as apple and pear [24–27]. Considering that overexpression of *GmFT2a* in both Arabidopsis and soybean promoted flowering in transgenic plants [37,38], we decided to integrate a *GmFT2a* expression cassette into CRISPR/Cas9 vector for gene editing, hoping that in addition to Arabidopsis, the construct generated may be used, in the future, for genome editing in soybean and possibly other plants.

**Fig 1:**
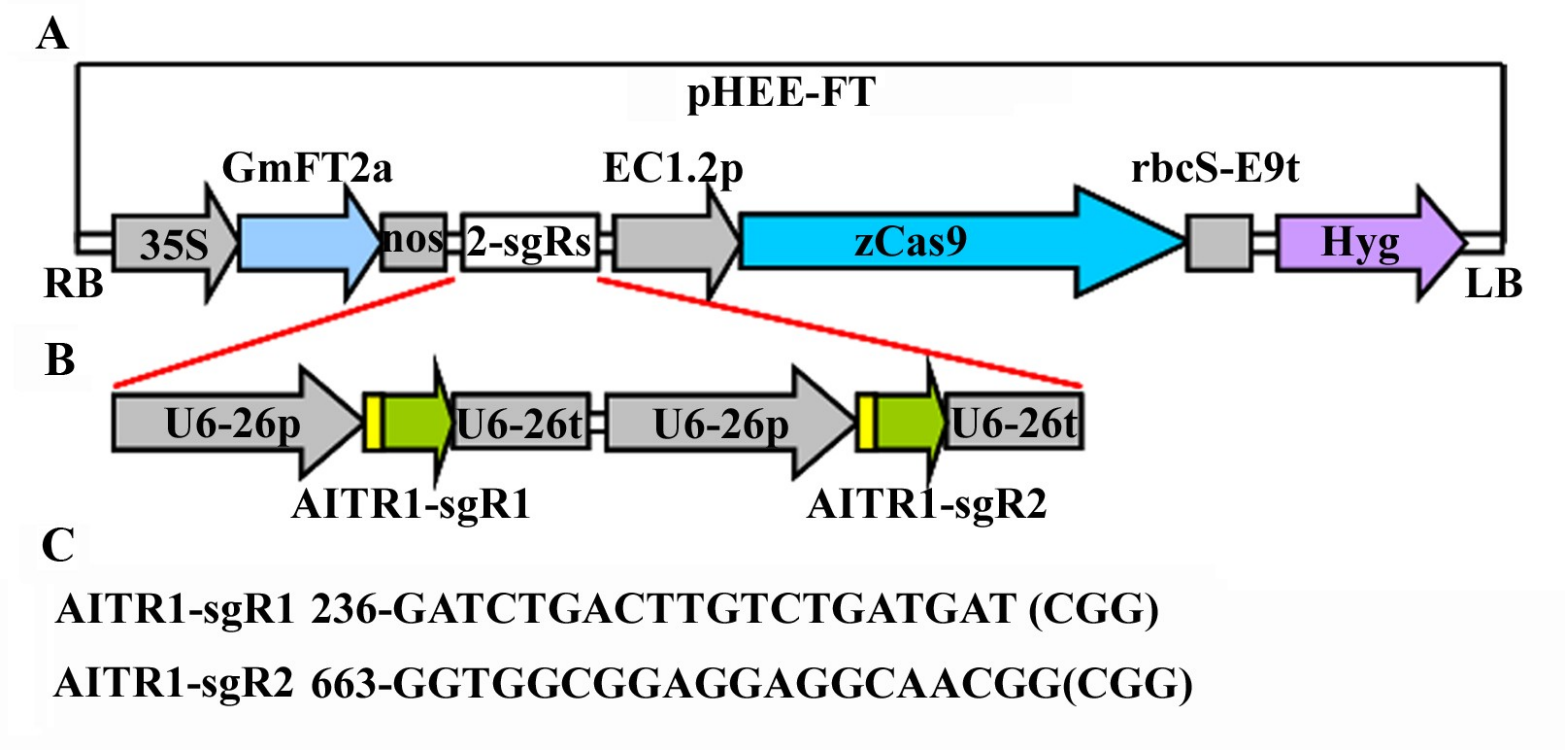
Generation of a *FT* expression cassette containing *pHEE* CRISPR/Cas9 construct for *AITR1* editing. (A) *pHEE* vector with a *FT* expression cassette. The full-length ORF sequence of *GmFT2a* was synthesized, and cloned into *pUC19* vector under the control of the *35S* promoter and terminated by the *nos* sequence. The *35S*:*GmFT2a-nos* cassette was amplified by PCR and then inserted into the *pHEE* vector at the *Pme*1 site by using Gibson assembly to generate *pHEE-FT* vector. (B) The *sgRNA* expression cassettes in the *pHEE-FT-AITR1* construct. The *sgRNA* sequences corresponding to the target sequences of *AITR1* were introduced into the *sgRNA* expression cassettes by PCR amplification, followed by Golden Gate reaction with the *pHEE-FT* vector. (C) Target sequences in *AITR1*. Numbers indicated the nucleotide position relative to the first nucleotide in the coding sequence of *AITR1*, PAM sites after the target sequences were indicated in the brackets.

The full-length coding sequence of *GmFT2a* was synthesized, and cloned into to the *pUC19* vector to generate the *35S*:*GmFT2a* construct. The whole *35S*:*GmFT2a-nos* expression cassette was then PCR amplified and cloned into the *pHEE* vector [33], at the *pme1* site to generate the *FT* expression cassette-containing CRISPR/Cas9 vector *pHEE-FT* (Fig 1A). In this vector, the *FT* expression cassette was inserted within the T-DNA fragment of the *pHEE-FT* vector as an independent expression cassette. The use of the strong double *35S* promoter to drive the expression of *GmFT2a* will enable the expression of *GmFT2a* at a high level in transgenic plants, therefore may lead to an easily visible early flowering phenotype in the transgenic plants.

### T1 transgenic plants generated using the *FT* expression cassette-containing CRISPR/Cas9 construct showed an early bolting phenotype

To examine if the genome editing using the *pHEE-FT* vector may enable fast generation and easy identification of transgene-free mutants, we made a *pHEE-FT* CRISPR/Cas9 genome editing construct to target *AITR1* (Fig 1B), a novel transcription factor gene that has been shown to regulate ABA signaling and abiotic tolerance in Arabidopsis [30], at two different target sites (Fig 1C).

**Fig 2:**
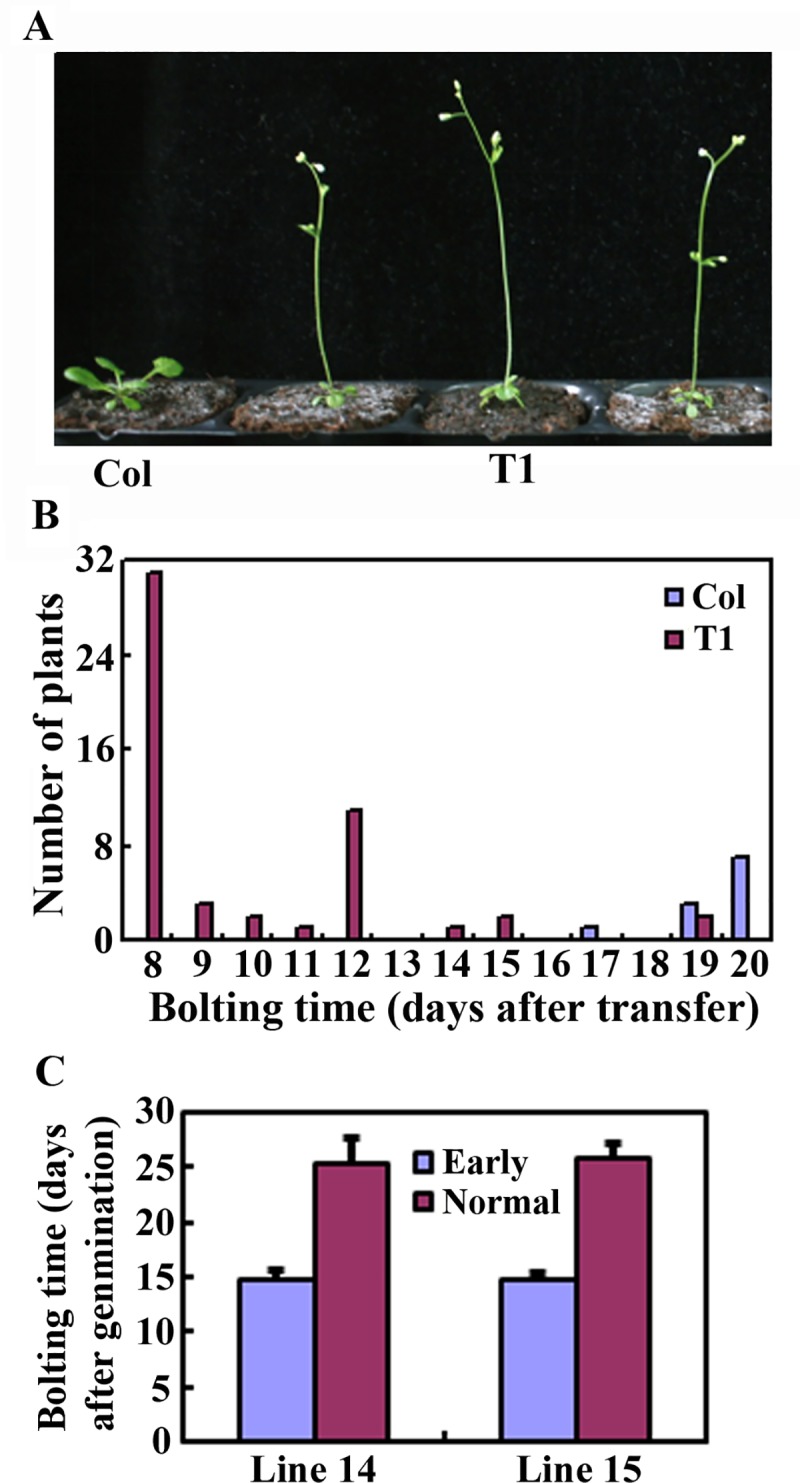
Early bolting phenotypes observed in the T1 and T2 transgenic plants. (A) Early bolting phenotype in some T1 transgenic plants. Transgenic plants were selected on antibiotic-containing 1/2 MS plates, and ~5-day-old transgenic seedlings were transferred into soil pots and grown in a growth room. As a control, seeds of Col wild type were germinated on 1/2 MS plates, and seedlings ~5-day-old were transferred into soil pots. Pictures were taken 10 days after the transfer. (B) Bolting time of the T1 transgenic plants. The date of bolting for the plants was recorded, and days after the transfer were calculated. For Col wild type plants, n = 11. For transgenic plants, n = 53. (C) Bolting time of the T2 transgenic plants from 2 independent T1 lines. T2 seeds were sown directly into soil pots and growth room. The date of bolting for the plants was recorded, and bolting time was calculated. Data represent mean ± SD of 9–40 plants.

After selected on antibiotic-containing plates, more than 70 T1 independent transgenic plants were obtained. After transferred into soil pots, some of them failed to survive. As expected, early bolting phenotype was observed in the transgenic plants (Fig 2A). Bolting was observed in transgenic plants as early as 8 days after the seedlings were transferred from the antibiotic-containing plates into soil pots, and the bolting time for these T1 transgenic plants ranged from 8–19 days after the transfer, whereas that for the Col wild type plants ranged from 17–20 days after the transfer (Fig 2B).

### Homozygous genome edited mutants were obtained in the T1 transgenic plants

The *pHEE-FT-AITR1* CRISPR/Cas9 construct was made to target two sites in *AITR1*, therefore fragment deletion should be expected in the transgenic plants if both sites can be edited. Because we intended to use early bolting phenotype as an assistant marker for transgene-free mutant isolation, transgenic plants with an early bolting phenotype, i.e., bolted 8 or 9 days after being transferred, were chosen for fragment deletion examination by PCR. Yet we included a few plants with medium or normal bolting time in this experiment to examine if this is a co-relationship between early bolting phenotype and genome editing status.

**Fig 3:**
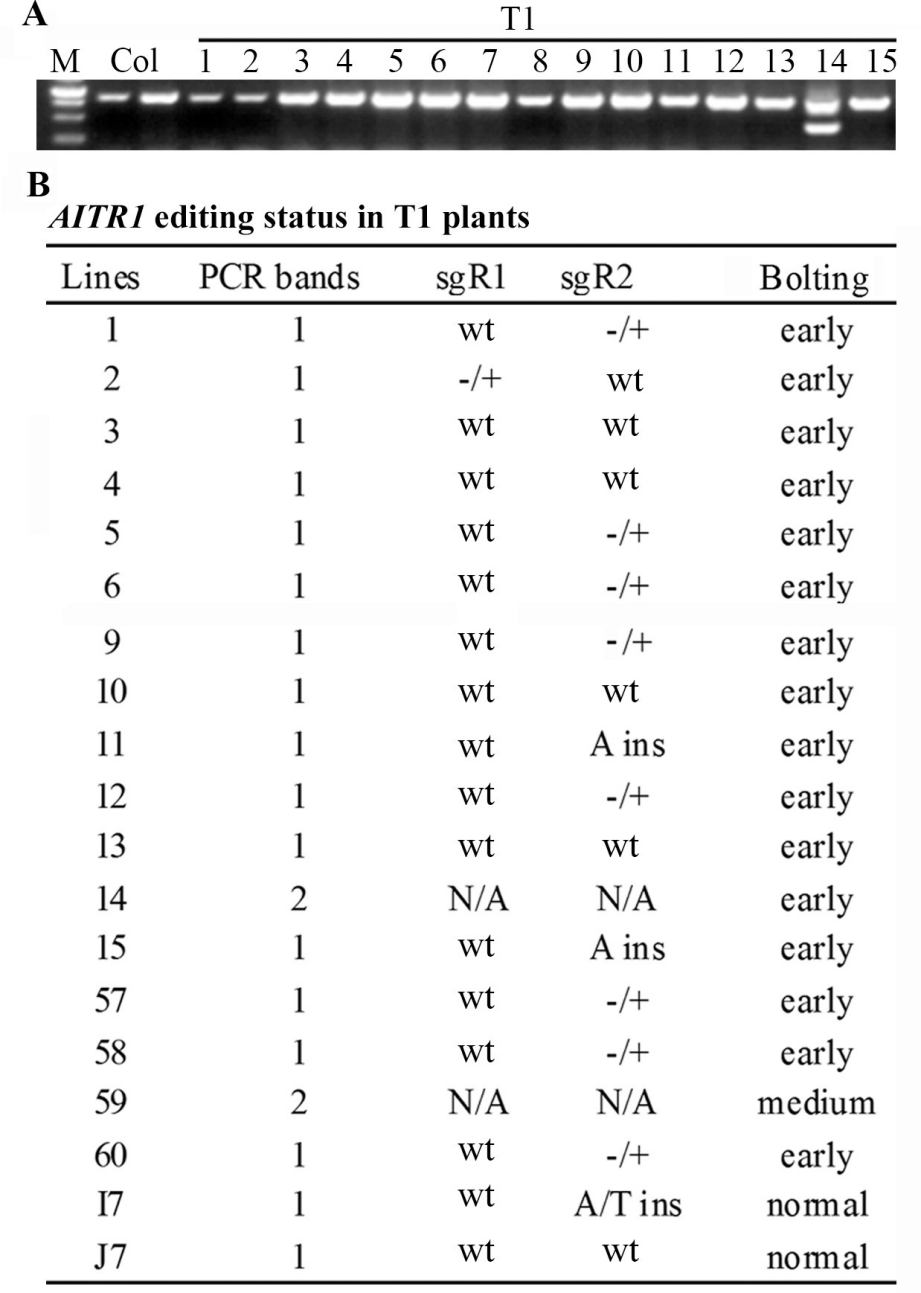
*AITR1* editing status in T1 transgenic plants. (A) PCR amplification of *AITR1* coding sequence in the T1 transgenic plants. DNA was isolated from leaves collected from Col wild type and individual T1 transgenic plants, and PCR was used to amplify coding sequence of *AITR1*. Amplification of *ACT2* was used as a control. Picture is image of PCR results for Col wild type and T1 transgenic plants lines 1 to 15, showing the 2 PCR product bands obtained in line 14, and 1 band for Col and other lines. M, 250 bp DNA maker. (B) *AITR1* editing status in sequenced individual T1 transgenic plants. PCR products were recovered from gel and sequenced. Sequencing results were examined and aligned with coding sequence of *AITR1* to check the editing status in the T1 transgenic plants. Early bolting lines were numbered sequentially, whereas lines normal bolting plants were numbered according to their initial location in the tray with a letter and a number. wt, not edited, -/+, edited but heterozygous, ins, homozygous or biallelic editing with nucleotide insertions as indicated, N/A, not sequenced.

Indeed, smaller PCR product band was obtained (Fig 3A). However, among about 50 T1 plants examined, only two plants, i.e., lines 14 and 59 with early and medium bolting phenotype, respectively yield two PCR bands, but none of them produced only one smaller band (Fig 3B). All other plants produced only one larger PCR band with expected site for the full-length coding sequence of *AITR1* (Fig 3A). These results suggest that both target sites in *AITR1* can be edited, but homozygous fragment deletion mutants were not obtained in the plants examined.

We therefore sequenced the full-length *AITR1* PCR products obtained from some of the plants, including 15 early bolting and 2 normal bolting plants that produced only one PCR product band, to see if we may get homozygous mutants with only one *AITR1* target site edited. Indeed, two of the early bolting plants, lines 11 and 15 were identified as homozygous mutants with a single nucleotide insertion at the second target site of *AITR1* (Fig 3B). One of the T1 plants with normal bolting phenotype, line I7 was edited at the second site with a single nucleotide insertion, but was a biallelic mutant (Fig 3B). We also found that another 9 early bolting plants sequenced were edited in one of the target sites but were heterozygous (Fig 3B). It should note that among the single site edited mutant, only one was edited at the first target site (Fig 3B).

### Homozygous genome edited transgene-free mutants were obtained in the T2 plants

Having shown that both homozygous and heterozygous mutants can be obtained in early bolting T1 transgenic plants (Fig 3), we decided to further examine whether transgene-free mutants can be easily obtained in T2 generation base on phenotypic observation. Four representative early bolting mutant lines, i.e., lines 6, 9, 14 and 15 were selected for the experiments, and T2 seeds were germinated directly in soil pots. Line 14 was chosen because PCR results indicated that both target sites of *AITR1* were edited in this line, where as line 15 was a homozygous, and lines 6 and 9 were heterozygous mutants based on sequencing results.

**Fig 4:**
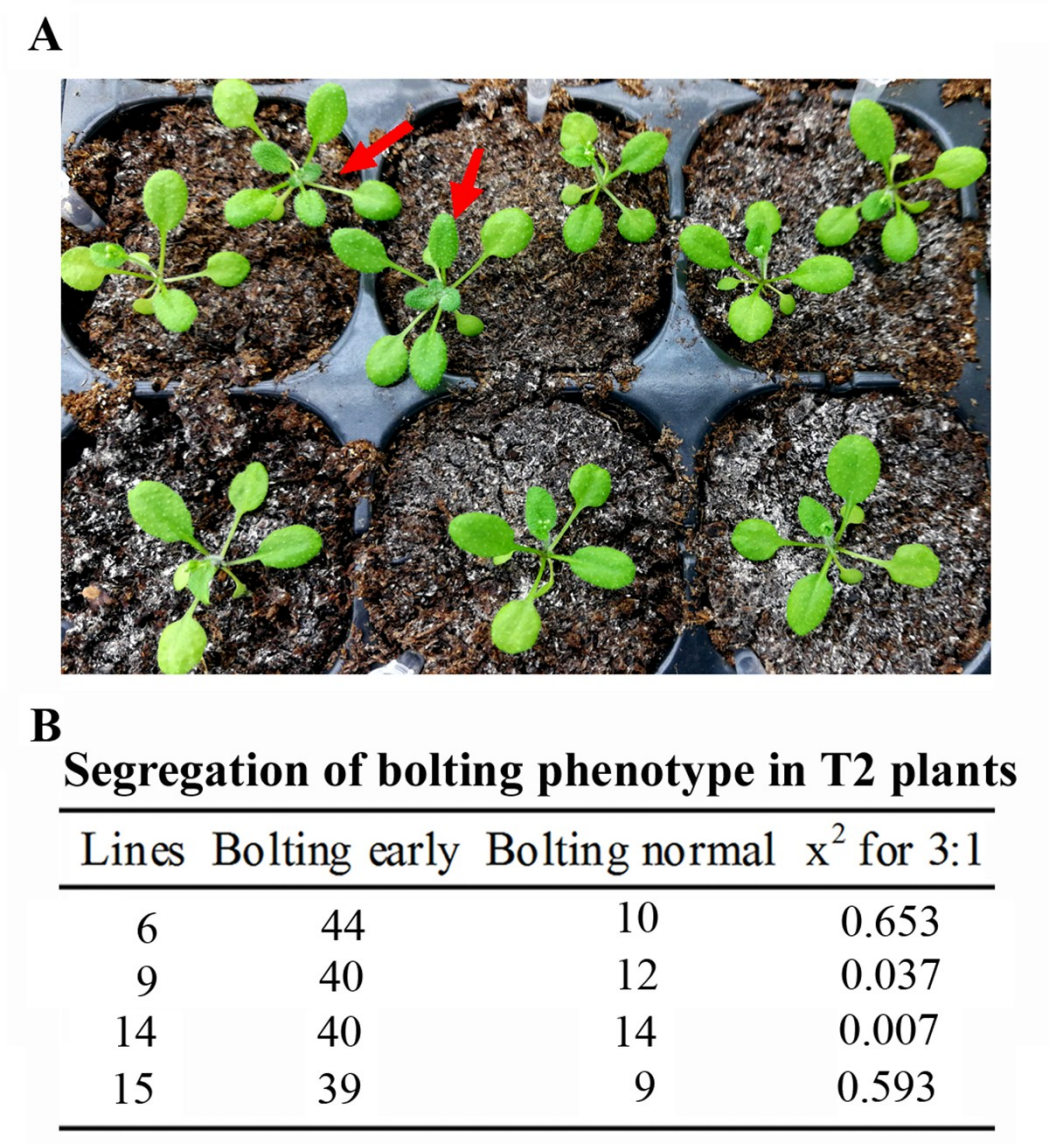
Phenotype segregation in the T2 generation of selected transgenic lines. (A) Bolting phenotype of the T2 plants from a single T1 transgenic line. T2 seeds were collected from selected T1 transgenic lines and sown directly into soil pots and grown in a growth room. Col wild type plants were generated and grown side by side with the T2 plants as a control. Pictures were taken 17 days after germination. Arrows indicate plants that did not bolt early. (B) Bolting phenotype segregation of the T2 plants from 4 T1 transgenic lines. Chi square analysis was performed on omni calculator (https://www.omnicalculator.com/statistics/chi-square).

Segregation on bolting phenotype was observed in T2 plants (Fig 4A). Multiple plants without early bolting phenotype were obtained for all four lines, and the segregation ratio for early and normal bolting plants was about 3:1 (Fig 4B). Quantitative analysis for lines 14 and 15 that the average bolting time were about 15 and 25 days after germination for the early and the normal bolting plants, respectively (Fig 2B).

**Fig 5:**
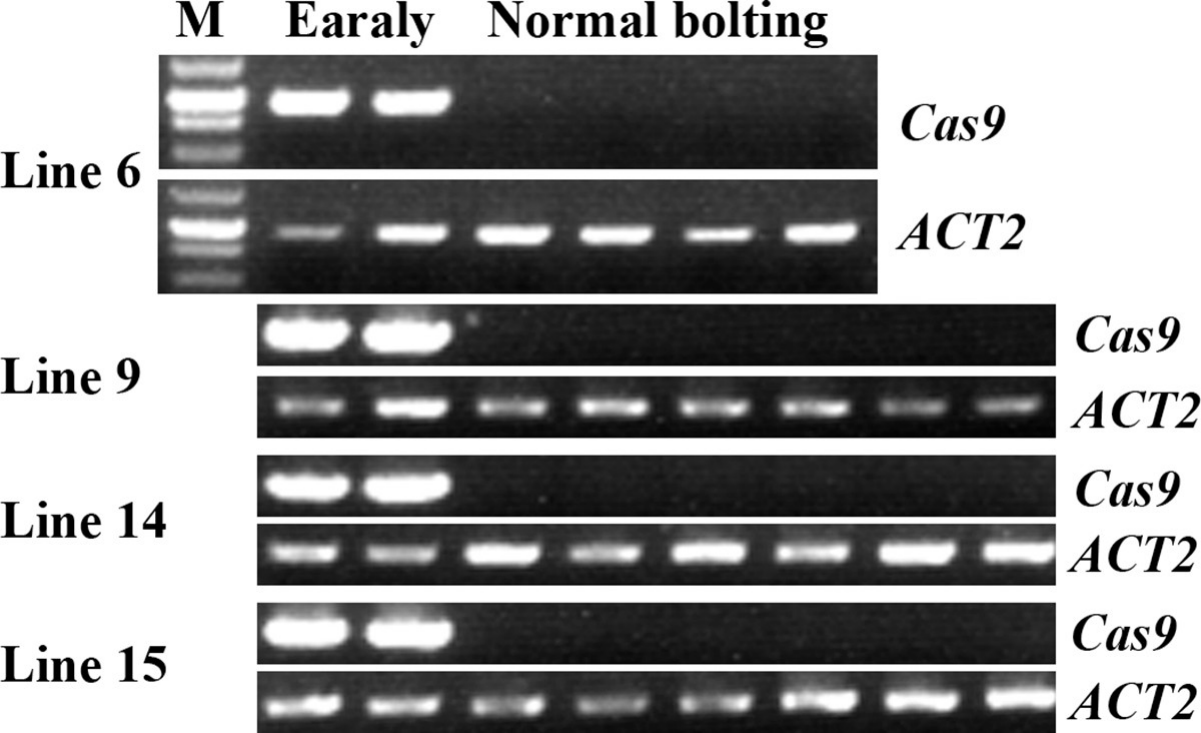
T2 plants with normal bolting time are transgene-free plants. DNA was isolated from leaves collected from 4–6 individual normal bolting T2 plants for each of the 4 lines, and PCR was used to amplify *Cas9* fragment. For each line, DNA was also isolated from two early bolting plants, and used a positive control for *Cas9* amplification. PCR amplification of *ACT2* was used as a control. M, 250 bp DNA maker.

We then examined if the plants without early bolting phenotype were transgene-free plants by amplification of *Cas9* gene. As shown in Fig 5, no PCR products were obtained in all the normal bolting plants examined, whereas PCR products were obtained in early bolting plants. These results suggest that transgene-free plants can be easily identified by bolting phenotype.

**Fig 6:**
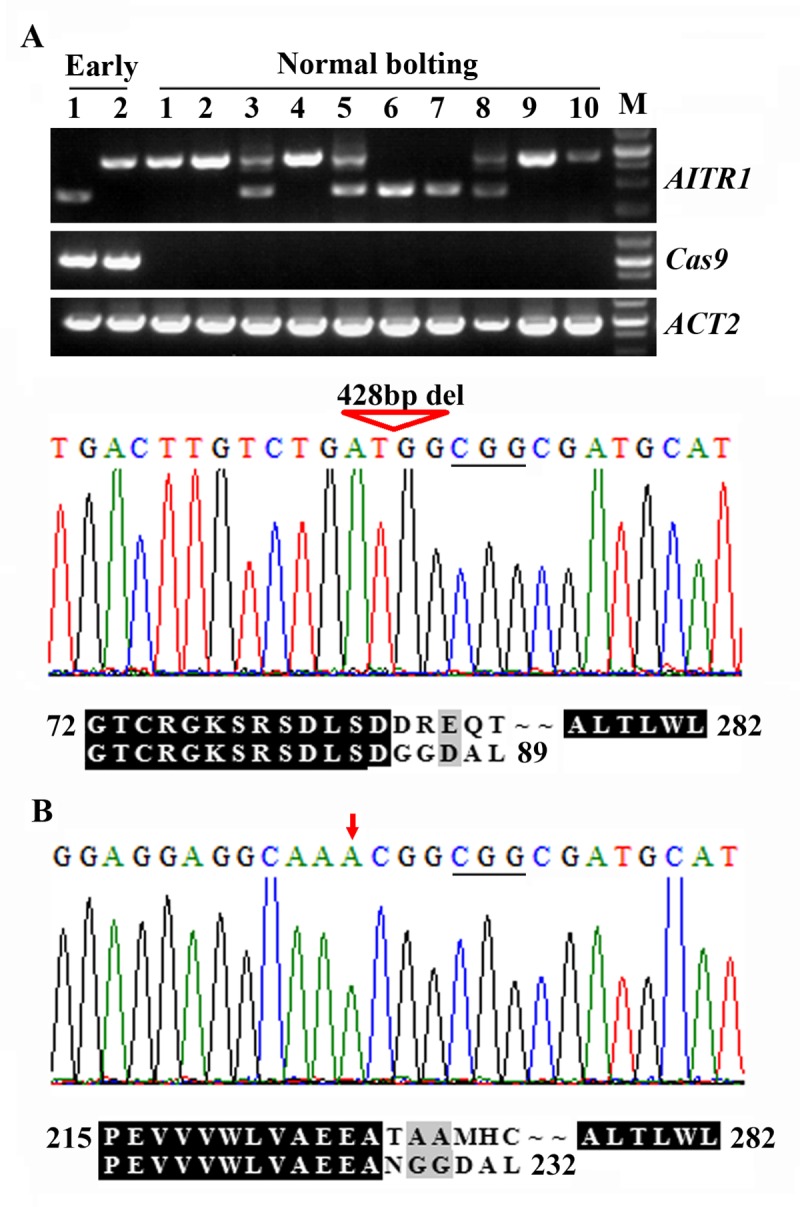
Isolation of genome edited transgene-free *aitr1* mutants. Transgene-free *aitr1* mutants isolated from line 14 (A) and lines 6, 9 and 15 (B). DNA was isolated from leaves collected from at least 2 individual normal bolting T2, and used as a template to amplify the coding sequence of *AITR1* and *Cas9*. Amplification of *ACT2* was used as a control. The PCR products were recovered from gel and sequenced. M, 250 bp DNA maker. Sequencing results were compared with coding sequence of *AITR1* to check the editing status. ORF of the *AITR1* sequences in the *aitr1* mutants were identified on ORFfinder (https://www.ncbi.nlm.nih.gov/orffinder/), and corresponding amino acid sequences were used for alignment with AITR1 amino acid sequences. Triangle indicates fragment deletion, arrow indicates nucleotide insertion, and underlines in the sequence results indicate the PAM sites. Numbers in the alignment indicate the position of amino acid relative to the first Met of AITR1.

By using PCR amplification, we found that two of the normal flowering plants in line 14 produced only a small band (Fig 6A), indicating that they were homozygous mutants. Sequence results showed that a 428bp fragment in *AITR1* was deleted in this mutant, leading to a few amino acid substitutions and premature stop after the 89^th^ amino acid residue (Fig 6A). One of the two randomly selected early bolting plants also produced a small band, however, as expected, it was not a transgene-free mutant (Fig 6A).

We then sequenced *AITR1* PCR products from two of the transgene-free plants produced by line 15 to confirm its homozygous genome editing status, and the normal bolting plants produced by lines 6 and 9 to identify homozygous mutants. We found that line 15 was indeed a homozygous mutant with a heritable single nucleotide insertion occurred in the second target site. We also successfully identified one homozygous mutant plant from line 6, and three from line 9. Interestingly, in all the four homozygous mutants identified from lines 6 and 9, the *AITR1* gene was edited the same way as that in line 15. In these homozygous mutants, the single nucleotide insertion in *AITR1* led to a few amino acid substitutions, and premature stop occurred after the 232^nd^ amino acid residue (Fig 6B).

## Discussion

**Fig 7:**
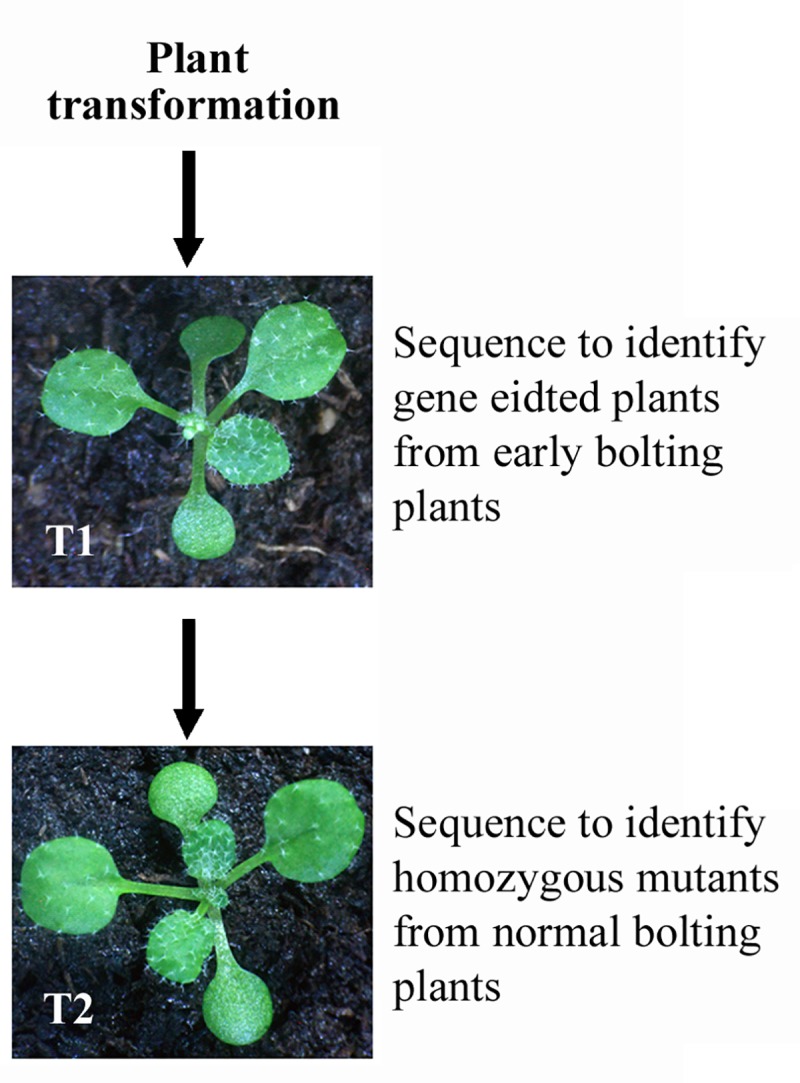
Simplified procedure for generating genome edited tansgene-free mutants by using *FT* expression cassette-containing CRISPR/Cas9 construct. Plants can be transformed and transgenic plants can be selected in a way similar to that for other constructs. Within the T1 transgenic plants, select plants with early-bolting phenotype, and sequence to examine genome editing status. Keep only genome edited plants. In the T2 plants germinated from seeds of the individual T1 plants, keep only those that bolt normally, and sequence to identify genome edited plants. These plants are genome edited transgene-free mutants. If necessary, confirm the transgene-free status by PCR amplification of *Cas9* fragment.

Removal of the Cas9 T-DNA from the transgenic plants is likely necessary to get stable and heritable mutants, expecially for commercial use of the genome-edited crops [7,10], whereas FT has been used to accelerate the plant breeding process by reducing juvenile phase of many plants [15,27,29]. By inserting the *GmFT2a* expression cassette into the *pHEE* CRISPR/Cas9 vector (Fig 1), we established a method for fast generation and easy identification of genome-edited transgene-free mutants in Arabidopsis (Fig 7).

In this method, the easily visible early flowering phenotype can be used as an indicator of plants with Cas9 T-DNA. In the T1 generation, only plants with early bolting phenotypes should be selected and genome editing status should be examined to identify mutations. In the T2 generation of the genome-edited T1 plants, only plants without early flowering phenotypes, i.e., transgene-free plants should be selected, and genome editing status should be examined to ensure that genome-edited transgene-free homozygous mutants will be obtained (Fig 7).

By using the *pHEE-FT* vector to generate a CRISPR/Cas9 genome editing construct for simultaneously targeting two sites in the ABA signaling and abiotic stress tolerance regulator gene *AITR1* [30], we successfully obtained genome-edited transgene-free *aitr* homozygous mutants (Fig 6). In the T1 generation, about two thirds of the early bolting plants examined have at least one target site edited (Fig 3), indicating that insertion of the *GmFT2a* expression cassette into the CRISPR/Cas9 vector did not affect the editing efficiency of Cas9. However, we noted that including the two plants with fragment deletion, only three plants had mutations at the first target site, suggesting that the two targets selected have different editing efficiency. Had both target sites had high editing efficiency, we should have been able to obtain homozygous fragment deletion mutants in T1 generation. Nevertheless, we obtained homozygous mutants with mutations that occurred at only one target site from the T1 plants (Fig 3), and we isolated transgene-free homozygous fragments deletion mutants in T2 generations (Fig 6).

Even though only the double-stranded DNA cleave based CRISPR/Cas9 genome-editing system was examined in this study, the concept used in this study may also be applied to the nucleotide substitution based CRISPR/Cas9 genome-editing system to facility transgene-free mutant isolation.

It should note that target site editing was also observed in T1 transgenic plants with medium or normal bolting time (Fig 3), suggesting that editing efficiency may not be positively correlated with early bolting phenotypes. This is likely because *FT* and *Cas9* in the vector were in two different expression cassettes, and were driven by different promoters (Fig 1), thus their expression levels may not always positively related in the transgenic plants. Consider that mutants can be obtained from early bolting T1 plants based on PCR results only or combined with sequencing (Fig 2), and transgene-free plants can be easily obtained from offsprings of the early bolting T1 plants based solely on bolting phenotype segregation (Fig 5), only T1 plants with early bolting phenotypes should be selected for next step analysis when using this method to generate for genome edited transgene-free mutants.

In addition to enabling easy identification of transgene-free mutants based on phenotypic observation, the early bolting phenotype also reduce the length of the juvenile phase, which led to reduced overall time length required for generating genome edited transgene-free mutants. In our case, the different bolting time between early bolting transgenic plants and Col wild type plants were more than 10 days even in soil grown T2 generations (Fig 2). Because early bolting plants were selected in T1, but plants with normal bolting time were selected in T2, the overall time length required for generating genome edited transgene-free mutants in Arabidopsis will be reduced by at least 10 days. In some cases, transgene-free mutants may not be able to be identified in T2 generations, and had to be identified on T3 generations. In that case, identifying mutants from offsprings of the gene-edited early bolting T2 plants may save more time than from that of the transgene-free heterozygous T2 mutants. However, it should be considered that early flowering Arabidopsis plants are weak, thus need to be taken good care of in order to obtain seeds. It should also be noted that because overexpression of *FT* promoted flowering, CRISPR/Cas9 vector with *FT* expression cassette is not suitable for editing genes involved in the regulation of flowering time in plants.

In this study, the effects of integration of *FT* expression cassette into CRISPR/Cas9 vector to accelerate transgene-free mutant isolation was tasted only in Arabidopsis, however, considering that the juvenile phase for most crops such as rice and soybean lasts for months, and that for most of the trees including fruit trees such as apple and pear lasts for years, whereas overexpression of *FT* greatly reduced the length of juvenile phase in most of the plants examined, including all the plants such as the model plants Arabidopsis, crops including rice and soybean, and fruit trees such as apple and pear [25–28,39–41], the method described here may benefit even more for genome editing based breeding for the plants with a long juvenile phase.

On the other hand, transgenic plants of different plant species may need to be selected in different antibiotics, and multiple genes may need to be edited simultaneously, by replacing the antibiotic gene and/or the *sgRNA* clone cassette, the *pHEE-FT* vector reported here may be used for genome editing for a single or multiple genes in different plant species.
